# Life-time expression of the proteins peroxiredoxin, beta-synuclein, PARK7/DJ-1, and stathmin in the primary visual and primary somatosensory cortices in rats

**DOI:** 10.3389/fnana.2015.00016

**Published:** 2015-03-04

**Authors:** Michael R. R. Böhm, Harutyun Melkonyan, Solon Thanos

**Affiliations:** ^1^Institute of Experimental Ophthalmology and DFG-Center of Excellence Cells in Motion (CiM), area C.4, School of Medicine, Westfalian-Wilhelms-University of MünsterMünster, Germany; ^2^Department of Ophthalmology, St. Franziskus Hospital MünsterMünster, Germany

**Keywords:** aging, cortex, peroxiredoxin, beta-synuclein, PARK7/DJ-1, stathmin

## Abstract

Four distinct proteins are regulated in the aging neuroretina and may be regulated in the cerebral cortex, too: peroxiredoxin, beta-synuclein, PARK[Parkinson disease(autosomal recessive, early onset)]7/DJ-1, and Stathmin. Thus, we performed a comparative analysis of these proteins in the the primary somatosensory cortex (S1) and primary visual cortex (V1) in rats, in order to detect putative common development-, maturation- and age-related changes. The expressions of peroxiredoxin, beta-synuclein, PARK[Parkinson disease (autosomal recessive, early onset)]7/DJ-1, and Stathmin were compared in the newborn, juvenile, adult, and aged S1 and V1. Western blot (WB), quantitative reverse-transcription polymerase chain reaction (qRT-PCR), and immunohistochemistry (IHC) analyses were employed to determine whether the changes identified by proteomics were verifiable at the cellular and molecular levels. All of the proteins were detected in both of the investigated cortical areas. Changes in the expressions of the four proteins were found throughout the life-time of the rats. Peroxiredoxin expression remained unchanged over life-time. Beta-Synuclein expression was massively increased up to the adult stage of life in both the S1 and V1. PARK[Parkinson disease (autosomal recessive, early onset)]7/DJ-1 exhibited a massive up-regulation in both the S1 and V1 at all ages. Stathmin expression was massively down regulated after the neonatal period in both the S1 and V1. The detected protein alterations were analogous to their retinal profiles. This study is the first to provide evidence that peroxiredoxin, beta-synuclein, PARK[Parkinson disease (autosomal recessive, early onset)]7/DJ-1, and Stathmin are associated with postnatal maturation and aging in both the S1 and V1 of rats. These changes may indicate their involvement in key functional pathways and may account for the onset or progression of age-related pathologies.

## Introduction

Cerebral maturation and aging is characterized by stereotypical structural and neurophysiological changes that result in marked variations in the dendritic morphology of pyramidal neurons in different cortical areas (Elston, 2002; Jacobs and Scheibel, 2002). Topologically, the cortical development of the visual and prefrontal cortex exhibit different time frames during the process of generating the dense network of pyramidal cells (Elston and Fujita, 2014). Moreover, there is a significant loss of synapses in the post-exhuberant postnatal cortex (Huttenlocher, 1990; Bourgeois and Rakic, 1993; Rakic et al., 1994; Huttenlocher and Dabholkar, 1997). These regional differences in pyramidal cell structure and synaptic density patterns may be the determinants of cortical function and may be associated with the functional and physiological aspects of learning processes for each cortical region (Elston, 2003; Spruston, 2008). The extent and branching complexity of dendrites, as well as the number and density of the spines on those dendrites, increases from the primary sensory area to higher-order processing areas, such as the prefrontal cortex (Elston, 2000; Jacobs et al., 2001; Elston et al., 2006a, 2009; Bianchi et al., 2013). In addition to neurons, neuroglia cells and microglia constitute the most numerous cell population in the mammalian brain (Mori and Leblond, 1969; Ling, 1976; Imamoto and Leblond, 1978; Shu and Richards, 2001; Rochefort et al., 2002). Neuroglia plays important roles in synapse formation during development, as well as in multiple forms of synaptic plasticity (Liu et al., 1994; Prewitt et al., 1997; Rochefort et al., 2002; López-Hidalgo and Schummers, 2014), whereas dynamic physical and molecular interactions between astrocytes and neurons control the morphology and structural plasticity of the dendritic spines. Once the appropriate synaptic connections are formed during the critical period of maturation and refinement, the function of astrocytes is likely modulated (Stevens, 2008).

At molecular level, the evolutionary conservation of gene expression in the aging brain includes increase in stress and inflammatory responses, and loss of mitochondria, neural plasticity, autophagy, and synaptic functions (Loerch et al., 2008; Bishop et al., 2010).

The frontal cortex as brain region with high-order cognitive functions, includes the motor and the somatosensory area. It receives less-coordinated activation with aging, suggesting global alterations in the integrative functions (Andrews-Hanna et al., 2007). In neurodegenerative disorders such as Alzheimer’s disease (AD), cognitive and mnestic alterations are predictors of age-related cortical function. (Huttenlocher, 1990; Bishop et al., 2010). This cognitive decline in frontal cortex is rather associated with alterations in synaptic connectivity than with neuronal loss and white-matter changes (Mattson and Magnus, 2006; Balietti et al., 2012). Physiological and anatomical alterations associated with aging are accompanied by changes in vision-associated qualities, including visual acuity, perception of contrast and wavelength, and impairment of binocular capabilities (Weale, 1975; Kline et al., 1983; Ross et al., 1985; Schefrin et al., 1999; Nomura et al., 2003; Mavroudis et al., 2012).

Most morphological studies of postnatal development have focused on the visual cortex due to its clearly demarcated characteristic anatomy, such as its very large granular layer (Huttenlocher et al., 1982; Huttenlocher, 1990). Timing and magnitude of growth, branching, spinogenesis and pruning in the basal dendritic trees of pyramidal cells differ dramatically among sensory, association, and executive cortical areas (Elston et al., 2009, 2010a,b, 2011b; Elston and Fujita, 2014). In primary (V1) and secondary visual cortex (e.g., V2, V3, and V4) the number of spines decreases in contrast to its increase in cytoarchitectonic areas in the posterior portion of the inferior temporal cortex (TEO) from early postnatal stages till adulthood (Elston et al., 2010a). Moreover, prolonged growth periods of dendritic trees in the primary auditory cortex (A1), anterior ventral portion of the inferior temporal cortex (TEav) and granular prefrontal cortex (Brodmann’s area 12) has been found compared to V1 (Cupp and Uemura, 1980; Anderson et al., 1995; Elston et al., 2009, 2011b). However, different growth profiles may reflect the type neuronal complexity and functional hierarchies in the adult brain (Jacobs and Scheibel, 2002; Elston, 2007; Spruston, 2008; Elston et al., 2009).

During aging a greater decline of complex pyramidal cells compared to simple cells has been found in the primary and secondary visual cortices of macaque monkeys (Liang et al., 2012). At morphological level, cerebral imaging has demonstrated the presence of atrophy of the visual cortex in aged humans (Park et al., 2004); those changes in vision-related areas of the brain include neurons (Satorre et al., 1985).

In addition to morphological alterations, the electrophysiological profile of pyramidal neurons also varies during postnatal development, maturation, and aging in different cortical layers, cortical areas, and species (Benavides-Piccione et al., 2002, 2006; Ballesteros-Yáñez et al., 2006; Elston et al., 2006b). Pyramidal cells in primary visual cortex (V1) show different electrophysiological profiles compared to granular prefrontal cortex and inferior temporal cortex (TE; Murayama et al., 1997; Amatrudo et al., 2012; Luebke et al., 2013). Those developmental differences in the electrophysiological signatures of pyramidal cells suggest different, specialized, and functional signatures of the adult cortex. Reflecting aging, measureable and significant losses of electrophysiological responses have been detected in the V1 of aged mammals (e.g., rats, cats, and New World monkeys) compared to their younger counterparts (Schmolesky et al., 2000; Hua et al., 2006; Wang et al., 2006). Moreover, different electrophysiological profiles have been found in primates in comparison to rodents during aging (McCormick and Prince, 1987; Kasper et al., 1994; Metherate and Aramakis, 1999; Zhang, 2004; Oswald and Reyes, 2008; Romand et al., 2011). The postnatal pyramidal cell development, spinogenesis, dendritic growth, and electrophysiological profiles varies considerably in different species and different cortical areas associated with visual processing (for review, see Elston and Fujita, 2014). However, perceptual deficits could not be explained by morphological alterations in the retina alone, and are probably only related to cortical areas (Satorre et al., 1985; Ahmad and Spear, 1993; Kim et al., 1996).

In a previous comparative proteomic analysis of the neuroretinas of marmosets and rats, we found that peroxiredoxin (Prx), beta-synuclein (SNCB), Parkinson’s disease (PD; autosomal recessive, early onset) 7/DJ-1 (DJ-1), and stathmin (STMN) were regulated in an age-related fashion in both species (Böhm et al., 2013). The aim of the present study was to explore the comparative expression characteristics of Prx, SNCB, DJ-1, and STMN in the primary somatosensory cortex (S1) and primary visual cortex (V1) of the rat. We presumed that significant regional differences were present in the expressions of the aforementioned proteins with respect to level of maturation and age between both the frontal and visual cortical areas. The rationale for using S1 and V1 was that these areas receive quite different afferent input, serve distinctly different functions, are topologically the most distant areas within the cortex, and show high divergence of pyramidal neuron morphometry together with the differential synaptic densities in the different cortical regions (e.g., prefrontal and somatosensory) cortex compared to the visual cortex (Geschwind and Rakic, 2013; Elston and Fujita, 2014). The distribution profiles and cellular localizations of the selected proteins are demonstrated herein, assigning them a generalized role in the processes in which they are involved in the brain.

## Materials and methods

### Animals

All animal work was conducted under the guidelines of the Animal Welfare Act and under the oversight and approval of the University and Governmental Institutional Animal Care and Use Committee (LANUV-NRW, Permission numbers 8-87-50.10.46.09.018 and 8-87-50.10.36.09.068 for rats). Sprague-Dawley rats were housed in standard animal rooms under a 12-/12-h light/dark cycle, with food and water provided ad libitum. In total, 72 rats (24 neonatal and 48 adult and/or elderly animals) were used, covering the following ages: postnatal day (P0) (i.e., the day of birth) (*n* = 24 animals), the young-adult stage (adolescence)—6 months after birth (6 m) (*n* = 12 animals), middle-age—12 and 18 months after birth (12 m and 18 m, respectively) (*n* = 12 animals for each group), and aged (elderly)—30 months after birth (30 m) (*n* = 12 animals).

### Brain dissection

Protein and RNA extraction were achieved following microdissection of the selected cortical regions, according to Palkovits et al. (Palkovits and Brownstein, 1988; Chiu et al., 2007). In brief, the animals were euthanized and the brain was removed from the skull and rinsed in ice-cold diethylpyrocarbonate-treated Milli-Q water to remove any surface blood. The brain was then placed onto a cold metal plate and the right and left hemispheres were separated. The olfactory bulb was dissected out and discarded. Both areas of interest are well dissectible when the cortex is viewed from the dorsal aspect. To ensure that in both cases and in relation to different maturated brains the areas of interest are included, the parietal part of the most anterior pole was dissected to include S1 (respectively the primary somatosensory area), whereas the cranial part of the most occipital pole was dissected to include V1 (respectively the primary visual area). The barrel cortex was not considered in preparation of S1 in this study (Woolsey and Van der Loos, 1970; Woolsey et al., 1975). The selected region was flash frozen in liquid nitrogen and stored at –80°C until use.

### Western blotting

Probes (P0, *n* = 8, 6–30 m, *n* = 4 for each group) obtained from either visual and frontal cortices were subsequently lysed in RIPA buffer [containing 0.1% sodium dodecylsulfate (SDS)] with additional protease inhibitor cocktail (Roche, Mannheim, Germany) and 1mM phenylmethylsulfonyl fluoride (Sigma-Aldrich), followed by further trituration and ultrasound treatment. The samples were sonicated and heated, and then the protein concentrations therein were determined using Bradford reagents (Bio-Rad, München, Germany). The samples were then transferred to SDS sample buffer containing 130 mM Tris-HCl (Carl Roth, Karslruhe, Germany), 10% w/v SDS, 10% mercaptophenol, 20% glycerol, and 0.06% w/v bromophenol blue (all Sigma-Aldrich). Fifty micrograms of protein from each sample were fractionated on 12% and 14% SDS–polyacrylamide gels (depending on the molecular weight of the target protein) with a protein marker (Bio-Rad, Hercules, CA, USA). After electrophoresis, the proteins were transferred onto a nitrocellulose membrane (Whatman, GE Healthcare Europe, Freiburg, Germany). The blots were incubated in blocking solution containing 5% fat-free dried milk (Carl Roth) and 0.1% Tween-20 PBS (Sigma-Aldrich) for 1 h, followed by incubation overnight at 4°C with a primary antibody, as listed in Table 1. The control antibody, anticalnexin, was used at a dilution of 1:10,000. The membrane was then incubated with a horseradish-peroxidase-conjugated secondary antibody (Sigma-Aldrich) in blocking solution for 1 h at room temperature. Antibodies were detected by enhanced chemiluminescence (Amersham, Rockville, MD, USA), and the relative densities of the protein spots were analyzed using Alpha Ease (Alpha-Ease FC software 4.0, Alpha Innotech, Biozym Scientific, Vienna, Austria). The protein density of a fixed area was determined for each spot after subtracting the specific background density in the surrounding region. The spot density was correlated and corrected against the relative density of the particular application control. The spot density of the samples from the P0 group was defined as the respective reference values, and the relative values at the other ages were calculated. In cases of missing or sparsely expression levels in neonatal ages, those obtained at 6 m were defined as reference values. Means and standard deviations of the relative relationships of the proteins were obtained for gels of four individual samples, each run three times for each individual group. The data are presented as mean ± SD values. The primary and secondary antibodies used are listed in Table 1.

**Table 1:** **Primary and secondary antibodies used for Immunohistochemistry and Western-blot**.

Immunohistochemistry				
1st Antibody	Species	Solution	Company
Peroxiredoxin (1–4)	Rabbit polyclonal	1:100	Santa-Cruz
DJ-1/Park7	Rabbit polyclonal	1:100	Abcam
Beta-Synuclein	Rabbit polyclonal	1:200	Abcam
Stathmin 1	Rabbit polyclonal	1:500	Abcam
NF-200	Mouse monoclonal	1:200	Sigma
GFAP	Mouse monoclonal	1:250	Sigma
CD11b/c (OX-42)	Mouse monoclonal	1:50	Serotec
**2nd Antibody**	**Species**	**Solution**	**Company**
(Cy)-2	Goat—Anti-mouse	1:400	Jackson Immunoresearch
TRITC	Goat—Anti-rabbit	1:400	Sigma
**Western blot**				
**1st Antibody**	**Species**	**Solution**	**kDa**	**Company**
Peroxiredoxin (1–4)	Rabbit polyclonal	1:4000	25 kDa	Santa-Cruz
DJ-1/Park7	Rabbit polyclonal	1:6000	20/24 kDa	Abcam
Beta-Synuclein	Rabbit monoclonal	1:2000	14 kDa	Abcam
Stathmin 1	Rabbit polyclonal	1:1000	17 kDa	Abcam
**2nd Antibody**	**Species**	**Solution**	**kDa**	**Company**
Calnexin	Rabbit polyclonal	1:10000	90 kDa	Sigma

*DJ-1, PARK [Parkinson disease (autosomal recessive, early onset)] 7/DJ-1; NF-200, high-molecular-weight neurofilament; GFAP, glial fibrillary acidic protein; CD11b/c, cluster of differentiation molecule 11b/c; (Cy)-2, cyanine; TRITC, tetramethylrhodamine; kDa, kilodalton*.

### Quantitative reverse-transcription polymerase chain reaction

RNA isolation from probes (P0, *n* = 8, 6–30 m, *n* = 4 for each group) was achieved using the RNeasy kit (Sigma-Aldrich) according to the protocols provided by the manufacturer. The results were quantified using a UV/visual spectral photometer (NanoDrop ND-1000, Peqlab, Erlangen, Germany). cDNA was synthesized from 1 μg of total RNA using the High-Capacity cDNA Reverse Transcription Kit from Applied Biosystems (Darmstadt, Germany). The quantitative polymerase chain reaction (PCR) primer pairs were designed for SYBR-Green-based real-time quantitative reverse-transcription polymerase chain reaction (qRT-PCR). The following primers were used in the PCR analysis:

**Peroxiredoxin (Prx) 2**: (NM_017169.1):

forward, CATGGCCTCCGGCAA;

reverse, AAAGGCACCATCCACCACGGC

**Beta-Synuclein (SNCB)**: (NM_031686.1):

forward, GGGCCTTGTCCCATTCACGGC;

reverse, TGCCTGCTTCTATATCCCGGCTTGG

**PARK [Parkinson disease (autosomal recessive, early onset)]7/DJ-1 (DJ-1)**: (NM_057143.1):

forward, ACCGCGCAGGAAAAACACGC;

reverse, CTGCCAGACGGCTCTGCAC

**Stathmin (STMN) 1**: (NM_017166.1):

forward, TCCGAGCCGCCTGGCTTAGG;

reverse, GTCCCGTGTCCCCGGCTAGG

qRT-PCR was performed using the SYBR Green PCR kit (Applied Biosystems) according to the protocols provided by the manufacturer. Relative protein expressions were calculated as 2–ΔCt^specific gene^/2–ΔCt^mean (housekeeping genes)^, using glyceraldehyde phosphate dehydrogenase as an endogenous housekeeping control gene. For relative quantification (RQ), the comparative Ct (Δ–ΔCt) method was employed; the results are presented relative to the expression level at P0. The data are presented as mean ± SD values.

### Immunohistochemistry

The regional localization and cellular expression of Prx (1–4), SNCB, DJ-1, and STMN in cortical cells was explored using immunohistochemistry (IHC). The animals (P0, *n* = 8, 6–30 m, *n* = 4 for each group) were given a lethal overdose of anesthesia induced using a mixture of 10% ketamine (Ceva Sanofi, Düsseldorf, Germany) and xylazine (Cefa Sanofi), and then perfused transcardially with phosphate-buffered saline (PBS; Sigma-Aldrich, Taufenkirchen, Germany) followed by 500 ml of a fixative containing 4% paraformaldehyde (PFA, pH 7.4) in 10 mM PBS. The brain of each animal was then extracted and postfixed in 4% PFA for 24 h. Fixed samples were processed in an automated tissue processor (Bavimed Histomaster 2062-DI 2L, Birkenau, Germany) and then transferred directly into 70% ethanol for 8 h prior to initiation of processing, followed by incubation for 15 min in each of the following: 99% ethanol, 96% ethanol, xylene (×2), and paraffin (×2). They were then infiltrated with low-melting-point paraffin wax (Sasol Wax, Hamburg, Germany) for 1 h at 56°C three times, with each brain being embedded separately. The paraffin-embedded brains were kept dry at 4°C until use.

The paraffin-embedded brains were sectioned coronally at a thickness of 4 μm using a microtome (CM 1500, Leica, Bremen, Germany), and mounted onto glass slides. The procedure for selecting slices of the region of interest is described briefly further below in the method section.

Before immunohistochemical staining, the selected slides/sections were warmed (at 60°C for 30 min) and then deparaffinized by soaking them twice in xylene for 10 min each time (Panreac Appli-Chem, Darmstadt, Germany), followed by two 3-min drenches in 99%, 96%, and 70% ethanol, followed by distilled water. After rinsing the slides with PBS (2 × 5 min), the sections were incubated for 30 min with blocking solution containing 10% goat serum (Sigma-Aldrich) for 2 h at room temperature, and then with primary antibodies overnight at 4°C. After washing, the sections were incubated with secondary antibodies (1 h at room temperature), washed in PBS, and then coverslipped with antifade mounting medium (Mowiol, Hoechst, Frankfurt, Germany) containing 4’,6-diamidino-2-phenylindole to stain the cell nuclei. Primary and secondary antibodies are provided in Table 1.

### Verification and identification of Prx, SNCB, DJ-1, and STMN in various cortical cell types

Differences in the expressions of Prx, SNCB, DJ-1, and STMN in different cortical cell types were analyzed by immunohistochemical staining of both the frontal and visual cortices. First, selected slices were routinely stained with hematoxylin and eosin (H&E) for basic morphological evaluation including comparison with the rat brain maps illustrated by Swanson (1992). The primary somatosensory cortex (S1) and primary visual cortex (V1) were correctly localized on examination of selected H&E-stained slides with bright-light microscopy (data not shown). Slices of selected regions were then immunohistochemically stained as described above.

Each section was stained with both an antibody detecting one of the cortical cell types—high-molecular-weight neurofilament (NF-200) for neuronal cells, glial fibrillary acidic protein (GFAP) for glial cells, or cluster of differentiation molecule 11b/c (OX-42) for brain microglia– and an antibody detecting Prx, SNCB, DJ-1, or STMN (Nadeau and Rivest, 2000). Colocalization of a cell-type-specific antibody with a specific protein antibody indicates expression of that particular protein in that particular cell type. Negative controls comprised sections that were processed as for the other sections but without the addition of a primary antibody. Control and experimental sections were stained simultaneously to avoid variations in immunohistochemical staining. Primary and secondary antibodies are provided in Table 1.

The slides were viewed with the appropriate filter on a microscope equipped with epifluorescence (Apotome 2, Carl Zeiss, Jena, Germany) and appropriate software (ZEN 2012, Carl Zeiss). Z-stacks were applied and evaluated to identify the cellular localization of the proteins. The regions of interest were then analyzed qualitatively as follows. Digital images of three microscopic fields (non-overlapping) in each hemisphere of at least three independent samples were taken at a magnification of ×20 to enable quantification of the level of each of the selected cortical cells within layer IV (for pyramidal neurons), layer I (for glial cells, e.g., astrocytes), and layer I-V (for microglia) were distinguished from other layers by the presence of larger pyramidal neurons. The barrel cortex was not considered in studying S1 (Woolsey and Van der Loos, 1970; Woolsey et al., 1975). A counting box of dimensions 300 μm × 300 μm (a buffer zone of 10 μm on either surface was employed so as not to include cut surfaces and to spare out the edges) was superimposed onto the image to aid counting. The number of cortical cells obtained from DAPI merged images within the region of interest was counted with the cell count function of ImageJ software.^1^ Each of the automated counts was double checked manually, and cells were assessed only if the cell body was easily seen and had no secondary branches obscured by background staining or by another cell. Any specific cortical cell body that was immunopositive for the protein under analysis, independent of its intensity, was then counted manually.

The focus of these analyses was to reveal the relative number of double-labeled cells. These data were used to calculate the rates of costaining [% = (number of cell body positive for selected protein/number of cortical cell type × 100)] in the cortex of each age, as well as the means of the individual experimental groups. The data are presented as mean ± SD values. The method of counting and calculating the rates of costaining was performed according to He et al. (2014). Although ideally we would like to have estimated the total number of neurons, it was not possible to demarcate these frontal and visual-associated brain areas in order to estimate their entire volume, and so we relative neuron cell count was used to represent neuronal densities, as described previously.

### Data evaluation and statistics

All data regarding the means of specific costaining studies from IHC, optical density from Western blot (WB) analyses, and relative quantification from qRT-PCR studies were analyzed with a test for two independent samples (IBM SPSS Statistics 20.0) to examine conformity with the Gaussian distribution, and processed using the ANOVA (for a Gaussian distribution) and Friedman test (for a non-Gaussian distribution). Local *p* values were corrected for multiple comparisons using the Holm-Bonferroni method. *p*-Value < 0.05 were judged as statistical significant. Figures were prepared with image-processing software (Photoshop, Adobe Systems, San Jose, CA, USA), and the overall brightness and contrast were adjusted without retouching.

## Results

First, the expressions of Prx, SNCB, DJ-1, and STMN in the S1 and V1 were verified at the protein and gene level using WB and qRT-PCR. Then, the cellular localization of the four proteins was examined immunohistochemically by double-staining S1 and V1 sections with antibody markers for three different cortical subsets of cells (neurons, glial elements, and microglial cells) and antibodies to Prx, SNCB, DJ-1, and STMN.

### Peroxiredoxin

#### Protein expression levels

**Figure 1:**
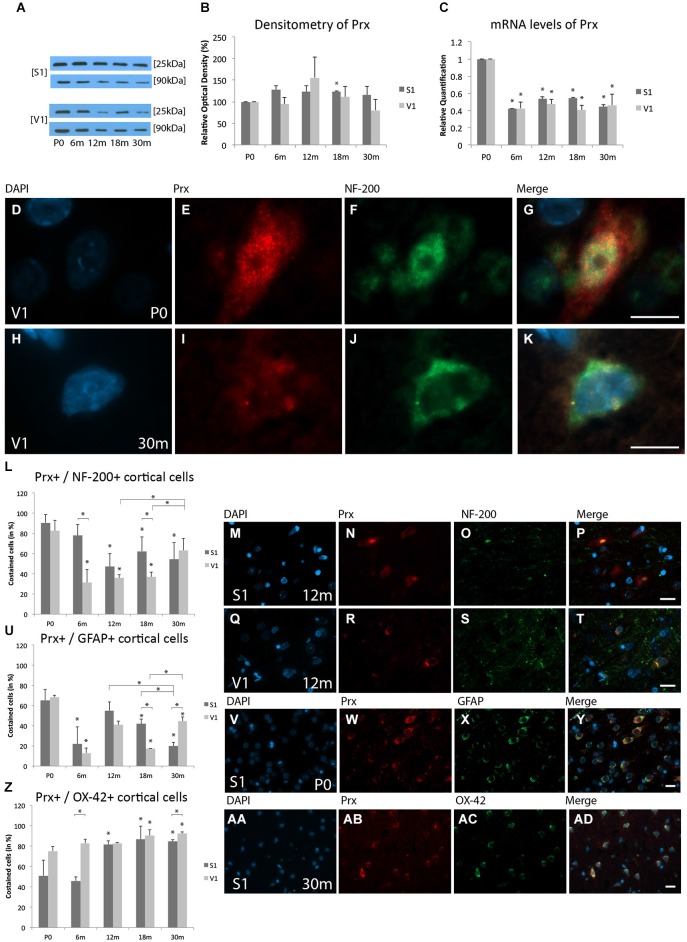
**Age-related expression and regulation of peroxiredoxins 1–4 (Prx) in the rat primary somatosensory cortex (S1) and primary visual cortex (V1). (A)** Western-blot analyses of cortex and **(B)** corresponding densitometric analyses of the Western-blot results relative to those measured at P0 (in %). Lysates of cortex treated as described in the main text were prepared and tested for Prx (25 kDa) expression. Calnexin expression verified the amount of protein loaded per lane. Protein bands are given in kilodaltons (kDa). **(C)** Quantitative reverse-transcription polymerase chain reaction (qRT-PCR) results for Prx mRNA levels relative to those measured at P0. Expression of cortical Prx (red) and several cortical cell types (green) revealed by immunohistochemical staining of 4-μm-thick sections of brain samples. **(D–K)** The intra- and extracellular localizations of Prx in neonatal (P0) and 30 months aged (30 m) V1 were detected in high-molecular-weight neurofilament (NF-200)-positive neuronal cells at higher magnification (×63). The rate of costained cells [%=(# of Prx+/cortical cell type+)/(# of cortical cell type+) × 100] (He et al., 2014) is shown with **(L–T)** NF-200 for neuronal cells in S1 and V1 at 12 m, **(U–Y)** glial fibrillary acidic protein (GFAP) for glial cells in S1 at P0, and **(Z,AA–AD)** cluster of differentiation molecule 11b/c (OX-42) for microglial cells in S1 at 30 m. The negative control was performed with cyanine (Cy)-2 and tetramethylrhodamine (TRITC) as secondary antibodies (data not shown). 4’,6’-Diamidino-2-phenylindole (DAPI) was used to stain the cell nuclei. Scale bars: **(D-K)**, 10 μm; **(M–P), (Q–T), (V–Y)**, and **(AA–AD)**, 20 μm. *Statistically significant difference at *p* < 0.05; *standing by itself mean statistically significant differences in relation to P0.

WB analysis of Prx expression at 6 m (S1, 128.5 ± 8.1%, *p* = 0.07; V1, 94.6 ± 15.5%, *p* = 0.6), 12 m (S1, 123.5 ± 13.8%, *p* = 0.3; V1, 156.0 ± 47.8%, *p* = 0.3), 18 m (S1, 123.1 ± 1.8%, *p* < 0.05; V1, 111.6 ± 23.6%, *p* = 0.5), and 30 m (S1, 116.1 ± 19.7%, *p* = 0.3; V1, 80.3 ± 25.2%, *p* = 0.5) revealed comparable expressions of Prx (25 kDa) in both cortical areas at all ages compared to P0 (Figures 1A,B). These data indicate that although there was a tendency toward changes in protein expression with age, the differences were not statistically significant.

#### mRNA expression levels

Prx mRNA level was significantly decreased at 6 m (S1, RQ = 0.42 ± 0.004, *p* < 0.05; V1, RQ = 0.43 ± 0.07, *p* < 0.05), 12 m (S1, RQ = 0.53 ± 0.03, *p* < 0.05; V1, RQ = 0.47 ± 0.06, *p* < 0.05), 18 m (S1, RQ = 0.54 ± 0.01, *p* < 0.05; V1, RQ = 0.41 ± 0.05, *p* < 0.05), and 30 m (S1, RQ = 0.45 ± 0.03, *p* < 0.05; V1, RQ = 0.46 ± 0.12, *p* < 0.05) compared to P0 (Figure 1C). These data show that Prx mRNA levels are decreased at all ages compared to P0, and confirmed the data observed at the IHC level.

#### Immunohistochemistry

The colabeling study revealed that Prx is expressed in neurons, glial cells, and microglia. Prx was localized mainly in the cytoplasm and in close association with the nucleus in NF-200-positive neuronal cells, GFAP-positive glial cells, and OX-42-positive microglial cells in the newborn cortex (Figures 1D–G). In the aged cortex, Prx was localized only in close association with the nucleus of neuronal cells and microglial cells (Figures 1H–K).

#### Neuronal staining

Prx was detected in neurons at all ages examined, and its distribution changed slightly with aging in both the S1 and V1. At the cellular level, at P0 Prx was costained with NF-200-positive neurons in both cortical areas (S1, 90.7 ± 8.1%; V1, 82.6 ± 10.6%; *p* = 0,2; Figure 1L). A decrease of these Prx-positive neuronal cells compared to P0 was found for both the S1 and V1 in the adult stages (e.g., 12 m: S1, 47.4 ± 12.9%, *p* < 0.05; V1, 35.9 ± 2.9%, *p* < 0.05; 18 m: S1, 62.4 ± 14.2%, *p* < 0.05; V1, 38.9 ± 4.6%, *p* < 0.05) compared to P0 (Figures 1L–T). The proportion of Prx-positive neurons continued to decline between the adult (e.g., 18 m) and elderly stages (30 m, 54.6 ± 16.3%, *p* = 0.57), and remained decreased compared to P0 (*p* < 0.05) in the S1. The proportion of Prx-positive neuronal cells was comparable between S1 and V1 in adult stages of age (12 m, *p* = 0.4; 18 m, *p* = 0.1). There was a tendency toward an increase in Prx-positive neurons at P30m in the V1 (63.4 ± 11.8%, *p* < 0.05) compared to adult stages (e.g., 18 m). No differences within the V1 between the ages 30 m and P0 were observed (*p* = 0.1) (Figure 1L).

#### Glial staining

At P0, comparable colabeling of Prx with GFAP-positive gial cells was found in both the S1 (65.3 ± 10.7%) and V1 (68.4 ± 2%, *p* = 0.8) (Figures 1U–Y). In the adult stages there was a decrease in glial Prx expression in both the S1 (18 m, 41.9% ± 4.9%, *p* < 0.05) and V1 (18 m, 17.5% ± 0.1%, *p* < 0.05) compared to P0. At 30 m (20.1 ± 3.4%), the proportion of Prx-positive glial cells had decreased further in the S1 compared to both the neonatal (20.1 ± 3.4%, *p* < 0.05) and the adult stages (12 m, 55.2% ± 8.4%, *p* < 0.05; 18 m, *p* < 0.05). In contrast, there was an increase in the proportion of Prx-positive glial cells in the V1 at 30 m (44.6 ± 4.0%,) compared to adult rats (12 m, 41.0% ± 3.7%, *p* = 0.3; 18 m, *p* < 0.05). There was a statistically significant difference between the S1 and V1 at 18 m (*p* < 0.05) and 30 m (*p* < 0.05) (Figure 1U).

#### Microglial staining

There was an increase in the proportion of OX-42-positive microglial cells colabeld with Prx in both cortical regions, with only slight differences between S1 and V1, beginning at P0 (S1, 51.0 ± 15.3%; V1, 75.2 ± 4.5%, *p* = 0.1) (Figure 1Z). Prx-positive staining of microglia increased with age, with intense staining being detected at 30 m in both cortical areas (S1, 84.7% ± 1.7%, *p* < 0.05; V1, 92.3 ± 1.4%, *p* < 0.05) compared to P0. There was a strong correlation of Prx staining in microglial cells and age in both cortical areas. An increased proportion of colabeled cells was present in the S1 at 12 m, 18 m and 30 m. A high colabeling rate of Prx and microglia was observed in the V1 in all stages of age (Figures 1AA–AD).

### Beta-synuclein

#### Protein expression levels

**Figure 2:**
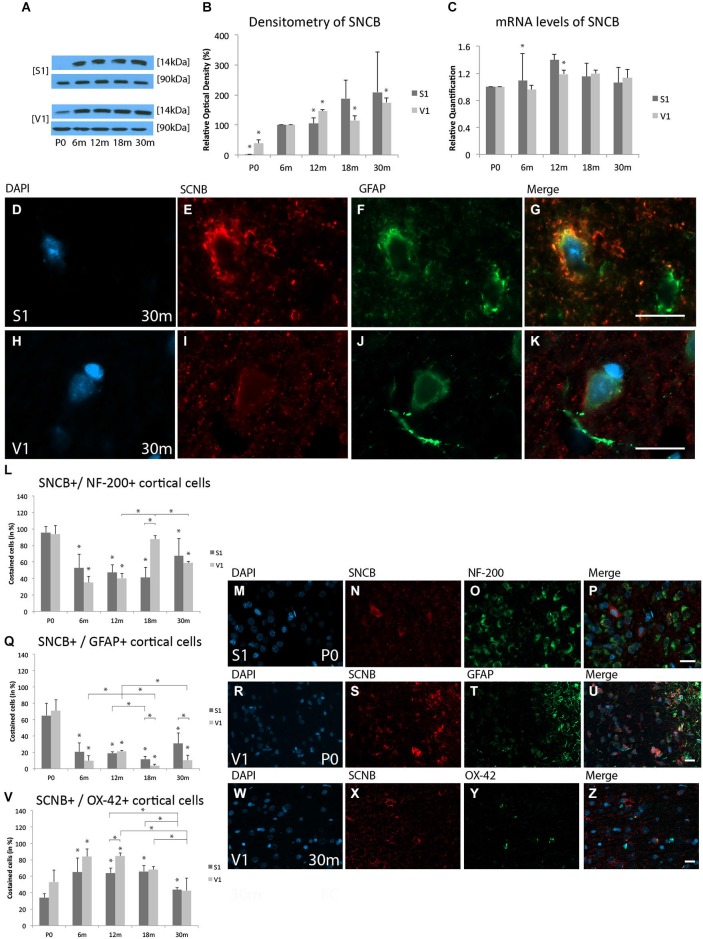
**Age-related expression and regulation of beta-synuclein (SNCB) in the rat S1 and V1. (A)** Western-blot analyses of cortex and **(B)** corresponding densitometric analyses of the Western-blot results relative to those measured at 6 months (6 m) of age (in %). Lysates of retinas treated as described in the main text were prepared and tested for SNCB (14 kDa) expression. Calnexin expression verified the amount of protein loaded per lane. Protein bands are given in kilodaltons. **(C)** Quantitative reverse-transcription polymerase chain reaction (qRT-PCR) results for SNCB mRNA levels relative to those measured at P0. Expression of cortical SNCB (red) and several cortical cell types (green) revealed by immunohistochemical staining of 4-μm-thick sections of brain samples. **(D–K)** Intra- and extracellular localizations of SNCB in aged (30 m) S1 and V1 were detected in GFAP-positive glial cells at higher magnification (63x). Rate of costained cells is shown in different cortical cell types: **(L–P)** Association between SNCB and NF-200-positive neuronal cells is demonstrated in S1 at P0. **(Q–U)** Colabeling of SNCB and GFAP-positive glial cells is shown in S1 at neonatal level. **(V–Z)** Costaining of SNCB and OX-42 was performed in V1 at 30 m. The negative control was performed with Cy-2 and TRITC as secondary antibodies (data not shown). DAPI was used to stain the cell nuclei. Scale bars: **(D–K)**, 10 μm; **(M–P), (R–U)**, and **(W–Z)**, 20 μm. *Statistically significant difference at *p* < 0.05. *standing by itself mean statistically significant differences in relation to **(B)** 6 m and **(C,L,Q,V)** P0.

Since SNCB was only sparsely detected in the S1 at P0, the expression at 6 m was used as a reference for optical density at later stages. The expression of SNCB was lower at P0 (S1, 1.33 ± 0.07%, *p* < 0.05; V1, 38.3 ± 13.3%, *p* < 0.05) than at 6 m. An increase in SNCB expression was detected in both the S1 (146.5 ± 4.4%, *p* < 0.05) and V1 (104.4 ± 19.4%, *p* < 0.05) at 12 m. There was no significant increase in SNCB expression in the S1 at 18 m (189.0 ± 61.0%, *p* = 0.06), while there was a significant increase in the V1 (115.5 ± 14.5, *p* < 0.05). Although no significant alterations in SNCB expression were detected in the aged S1 (209.5 ± 133.3%, *p* = 0.08), there was a significant increase in the aged V1 (175.3 ± 14.4%, *p* < 0.05; Figures 2A,B).

#### mRNA expression levels

SNCB mRNA levels were slightly up-regulated at 6 m in the S1 (RQ = 1.1 ± 0.04, *p* < 0.05), but remained unchanged in the V1 (RQ = 0.96 ± 0.4, *p* = 0.4). The greatest degree of SNCB mRNA up-regulation was found at 12 m in the V1 (RQ = 1.2 ± 0.09, *p* < 0.05); the slight up-regulation of SNCB mRNA detected in the S1 was not statistically significant (RQ = 1.4 ± 0.24, *p* = 0.07). The expression of this protein’s mRNA remained unchanged at 18 m (S1, RQ = 1.15 ± 0.16, *p* = 0.2; V1, RQ = 1.2 ± 0.2, *p* = 0.5) and 30 m (S1, RQ = 1.1 ± 0.25, *p* = 0.4; V1, RQ = 1.1 ± 0.23, *p* = 0.2) compared to P0 (Figure 2C).

These data show that the expression of SNCB increased significantly at both the protein and mRNA levels during maturation of the brain, and then remained stable after 6 m in the V1, and increased slightly after 18 m in the S1.

#### Immunohistochemistry

A faint staining for SNCB was observed in both the S1 and V1 in the neonate, with an overall increase with age. SNCB was localized to the cytoplasm of neuronal cells at younger ages, while in aged cortical glial cells SNCB was found in close association with the nucleus (Figures 2D–K).

#### Neuronal staining

Comparable SNCB-positive neuronal cells were found in neonatal rat cortices (S1, 95.9 ± 7.2%; V1, 93.9 ± 10.6%; *p* = 0.8; Figures 2L–P). Its proportion of costained cells decreased with age and remained stable until the adult ages in both the S1 (e.g., 6 m: 52.6 ± 20.4%, *p* < 0.05) and V1 (e.g., 6 m 35 ± 7.5%; *p* < 0.05). An increase of SNCB colabeled neuronal cells was detected in the V1 beginning at 18 m (87.5 ± 4.6%, *p* < 0.05; 30 m, 58.9 ± 1.9%, *p* < 0.05) compared to 12 m (40.3 ± 5.9%). The analyses revealed no change in neuronal SNCB staining in the S1 at this same stage (12 m, 47.3% ± 9%, *p* = 0.7; 18 m, 41 ± 12.5%, *p* = 0.4) compared to 6 m. This pattern changed after 18 m, with an increase in the proportion of SNCB-positive neurons with age up to 30 m (67.3 ± 20.8%), resulting in a distinct, but not significant increase of NF-200- and SNCB-positive cells in the S1 compared to adult stages of age (e.g., 18 m, *p* = 0.11; Figure 2L).

#### Glial staining

There was a direct correlation between SNCB and GFAP-positive cells at P0 in both the S1 (65.2 ± 14.9%) and V1 (70.9 ± 13.7%; *p* = 0.6; Figures 2Q–U). However, this close relationship between SNCB and glial cells decreased at 6 m (S1, 20.4 ± 11.1%, *p* < 0.05; V1, 9.7 ± 5.8%, *p* < 0.05) till elderly stages of age. A further decline in colabeling of SNCB in glial cells were found in both the S1 and V1 at 18 m (S1, 19 ± 1.7%, *p* < 0.05; V1, 3.4 ± 2.2%, *p* < 0.05) and in V1 at 30 m (10.2 ± 6.0%, *p* < 0.05) compared to 12 m (S1, 19% ± 1.7%; V1, 21.3 ± 2.3%) (Figure 2Q).

#### Microglial staining

In agreement with the finding of slight immunostaining for SNCB in microglial cells in both cortices at P0 (S1, 34.1 ± 4.5%; V1, 53.2 ± 14.3%; *p* = 0.08), there was an increase in the proportion of microglial cells positive for SNCB in both the S1 and V1 in the adult ages (e.g., 6 m: S1, 64.9%17.3%, *p* < 0.05; V1, 83.9 ± 9.4%, *p* < 0.05) (Figure 2V). This proportion then decreased in both cortices in elderly rats (30 m: S1, 43.9 ± 2.2%; V1, 42.7 ± 14.8%) compared to adult age stages (e.g., 12 m: S1, 64.1 ± 6.2%; *p* < 0.05; V1, 84.4 ± 4%;* p* < 0.05) (Figures 2V–Z).

### DJ-1

#### Protein expression levels

**Figure 3:**
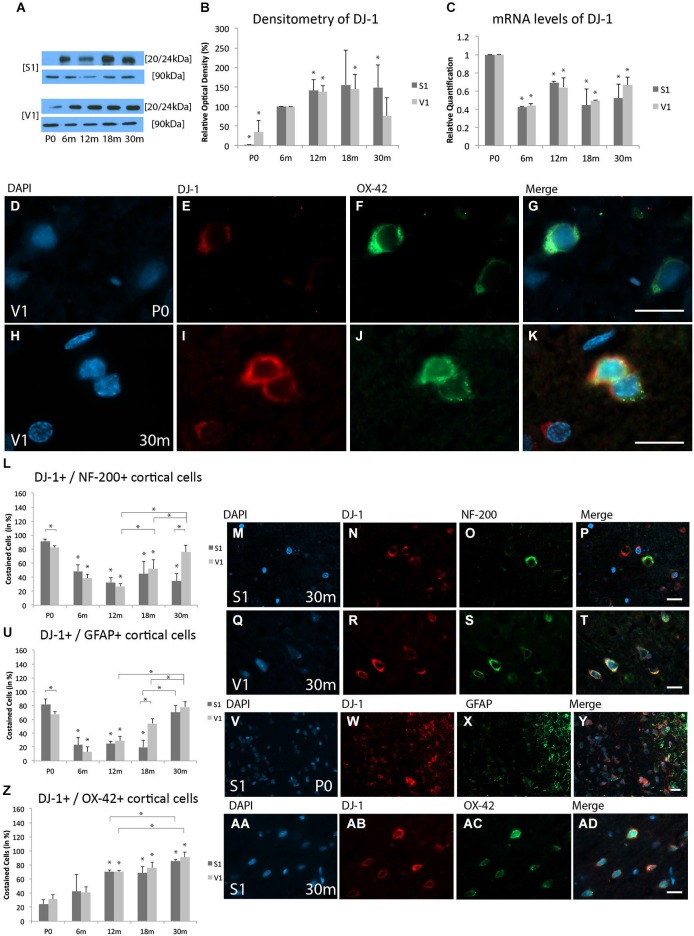
**Age-related expression and regulation of PARK[Parkinson disease (autosomal recessive, early onset)]7/DJ-1 (DJ-1) in the rat S1 and V1. (A)** Western-blot analyses of cortex and **(B)** corresponding densitometric analyses of the Western-blot results relative to those measured at 6 m (in %). Lysates of cortex treated as described in the main text were prepared and tested for DJ-1 (20/24 kDa) expression. Calnexin expression verified the amount of protein loaded per lane. Protein bands are given in kilodaltons. **(C)** Quantitative reverse-transcription polymerase chain reaction (qRT-PCR) results for DJ-1 mRNA levels relative to those measured at P0. Expression of cortical DJ-1 (red) and several cortical cell types (green) revealed by immunohistochemical staining of 4-μm-thick sections of brain samples. **(D–K)** The intra- and extracellular localizations of DJ-1 in neonatal (P0) and 30 months (30 m) aged V1 is shown in OX-42-positive microglial cells at higher magnification (×63). Costaining rate of DJ-1 is shown in different cortical cell types: **(L–T)** Association between DJ-1 and NF-200-positive neuronal cells is demonstrated at 30 m in S1 and V1. **(U–Y)** Costaining of DJ-1 and GFAP-positive glial cells was performed in S1 at P0. **(Z,AA–AD)** Colabeling of DJ-1 and OX-42-positive microglial cells is shown in S1 at P30m. The negative control was performed with Cy-2 and TRITC as secondary antibodies (data not shown). DAPI was used to stain the cell nuclei. Scale bars: **(D–K)**, 10 μm; **(M–P), (Q–T), (V–Y)**, and **(AA–AD)**, 20 μm. *Statistically significant difference at *p* < 0.05; *standing by itself mean statistically significant differences in relation to **(B)** 6 m and **(C,L,U,Z)** P0.

WB analysis revealed expression of DJ-1 in the V1 at all age stages. However, expression of DJ-1 was only sparsely detected in the neonatal S1. Compared to 6 m, there was a reduced expression of DJ-1 in both the S1 (1.4 ± 0.5%, *p* < 0.05) and V1 (34.8 ± 29.5%, *p* < 0.05). With increased age, significantly more DJ-1 was found in both cortices at 12 m (S1, 140.8 ± 27.3%, *p* < 0.05; V1, 137.4 ± 15.7%, *p* < 0.05). However, at 18 m increased DJ-1 expression was detected in the V1 (143.8 ± 39.1%, *p* < 0.05), but not in the S1 (155.5 ± 88.2%, *p* = 0.07). Increased expression of DJ-1 was detected in the aged (30 m) S1 (148.1 ± 58.6%, *p* < 0.05), but not in the V1 at the same age (76.1 ± 45.9%, *p* = 0.2; Figures 3A,B).

#### mRNA expression levels

DJ-1 mRNA levels were significantly decreased in both the S1 and V1 at 6 m (S1, RQ = 0.42 ± 0.01, *p* < 0.05; V1, RQ = 0.44 ± 0.02, *p* < 0.05), 12 m (S1, RQ = 0.69 ± 0.02, *p* < 0.05; V1, RQ = 0.64 ± 0.1, *p* < 0.05), 18 m (S1, RQ = 0.45 ± 0.2, *p* < 0.05; V1, RQ = 0.49 ± 0.004, *p* < 0.05), and 30 m (S1, RQ = 0.52 ± 0.5, *p* < 0.05; V1, RQ = 0.66 ± 0.09, *p* < 0.05) compared to P0 (Figure 3C). These data reveal a slight up-regulation of DJ-1 protein, but an overall reduction in its mRNA.

#### Immunohistochemistry

IHC for DJ-1 revealed stained cells in the S1 and V1 at all ages (Figures 3D–K). DJ-1 was localized to the cytoplasm of all cell types examined, including NF-200-positive neuronal cells, GFAP-positive glial cells, and OX-42-positive microglial cells in both the S1 and V1.

#### Neuronal staining

Colabeling of DJ-1 and NF-200-positive neurons was found at all ages (Figure 3L). Different costaining of DJ-1 and NF-200 was found in newborn rats in S1 (91.1 ± 3.6%) compared to V1 (82.1 ± 2.6%; *p* < 0.05; Figure 3L). There was a subsequent reduction in the proportion of DJ-1-positive neurons with increasing age (e.g., 18 m; S1, 45.1 ± 19.8%, *p* < 0.05; V1, 52.2 ± 12.4%, *p* < 0.05) compared to P0. There were no differences in the proportions of DJ-1-positive neurons between the adult ages (i.e., 6 m, 12 m) and cortical regions. The costaining of DJ-1 and NF-200-positive neurons was then unchanged between the adult ages (e.g., 18 m) and P30m in the S1 (34.4 ± 10.6%, *p* = 0.3), while an increase in the proportion of colabeled cells was found in the V1 (75.9 ± 9.7%, *p* < 0.05) compared to adult stages of age (e.g., 18 m) (Figures 3M–T).

#### Glial staining

Noticeable costaining of DJ-1 and GFAP-positive glial cells was detected in both the S1 (81.9 ± 7.4%) and V1 (67.6 ± 3.9%, *p* < 0.05) at P0 (Figures 3U–Y). However, there was a reduction in this association in both cortical areas at 6 m (S1, 23.7 ± 10.1%, *p* < 0.05; V1, 12.8 ± 7.5%, *p* < 0.05), followed by an increase, beginning in the S1 at P30m (18 m: 19.8 ± 10%, *p* < 0.05; P30m: 70.4 ± 0.1, *p* = 0.11) and in the V1 at 12 m (12 m: 28.8 ± 6.4%, *p* < 0.05; 18 m: 54 ± 6.8%, *p* < 0.05; P30m: 77.8 ± 7.9%, *p* = 0.07) compared to P0 (Figures 3U–Y).

#### Microglial staining

In contrast to the rare colabeling of DJ-1 and OX-42-positive microglial cells in both the S1 (24.5 ± 6.4%) and V1 (31.4 ± 6.4%) observed at P0 (Figure 3Z), a strong increase was detected at advanced ages, such as 30 m (S1, 85.2 ± 3%, *p* < 0.05; V1, 90.9 ± 7.3%, *p* < 0.05) (Figures 3Z,AA–AD).

### Stathmin

#### Protein expression levels

**Figure 4:**
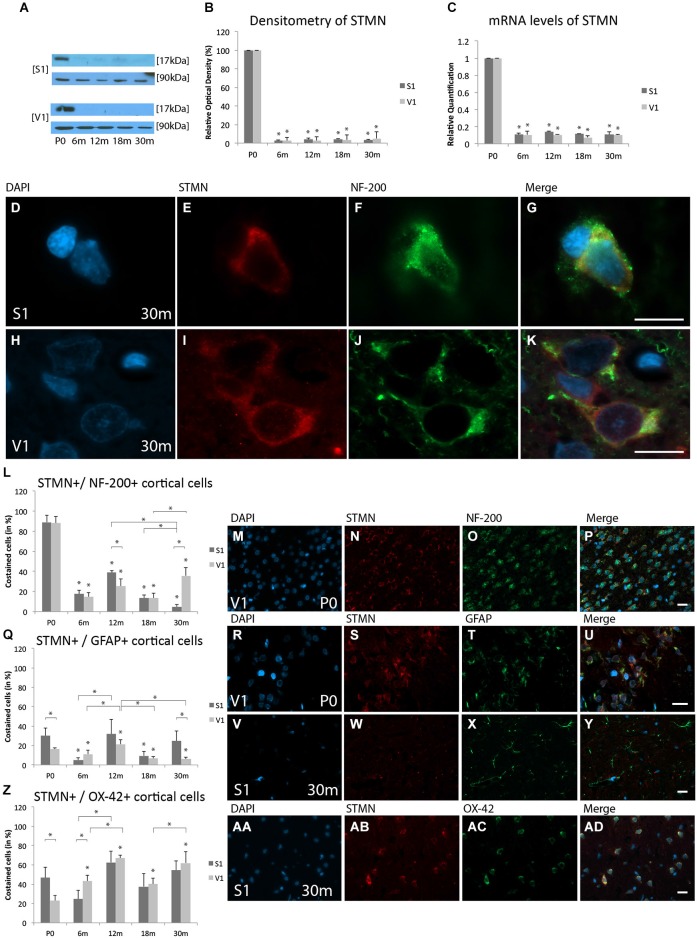
**Age-related expression and regulation of stathmin (STMN) in the rat S1 and V1. (A)** Western-blot analyses of retinas and **(B)** corresponding densitometric analyses of the Western-blot results relative to those measured at P0 (in %). Lysates of cortex treated as described in the main text were prepared and tested for STMN (17 kDa) expression. Calnexin expression verified the amount of protein loaded per lane. Protein bands are given in kilodaltons. **(C)** qRT-PCR results of STMN mRNA levels relative to those measured at P0. Expression of cortical STMN (red) and several cortical cell types (green) revealed by immunohistochemical staining of 4-μm-thick sections of brain samples. **(D–K)** Intra- and extracellular localizations of STMN (red) were detected in aged S1 and V1 in NF-200-positive cells (green) at higher magnification (×63). Costaining rate of STMN is shown in different cortical cell types: **(L–P)** Costaining of STMN is shown at P0 with NF-200-positive neuronal cells in the V1. **(Q–Y)** Association of STMN with GFAP-positive glial cells is demonstrated in V1 at P0 and S1 at P30. **(Z,AA–AD)** Colabeling of STMN with OX-42 is shown in S1 at 30 m. The negative control was performed with Cy-2 and TRITC as secondary antibodies (data not shown). DAPI was used to stain the cell nuclei. Scale bars: **(D–K)**, 10 μm; **(M–P), (R–U), (V–Y)**, and **(AA–AD)**, 20 μm. *Statistically significant difference at *p* < 0.05; *standing by itself mean statistically significant differences in relation to P0.

WB analysis revealed expression of STMN in both cortical regions at P0, with a massive decrease at 6 m (S1, 2.5 ± 0.7%, *p* < 0.05; V1, 2.5 ± 3.5%, *p* < 0.05), 12 m (S1, 3.9 ± 1.3%, *p* < 0.05; V1, 2.8 ± 3.9%, *p* < 0.05), 18 m (S1, 3.8 ± 1.3%, *p* < 0.05; V1, 3.6 ± 5.1%, *p* < 0.05), and 30 m (S1, 3.5 ± 0.5%, *p* < 0.05; V1, 5.0 ± 7.1%, *p* < 0.05) (Figures 4A,B).

#### mRNA expression levels

mRNA was significantly down-regulated at 6 m (S1, RQ = 0.11 ± 0.02, *p* < 0.05; V1, RQ = 0.10 ± 0.04, *p* < 0.05), 12 m (S1, RQ = 0.14 ± 0.01, *p* < 0.05; V1, RQ = 0.11 ± 0.0001, *p* < 0.05), 18 m (S1, RQ = 0.12 ± 0.002, *p* < 0.05; V1, RQ = 0.07 ± 0.02, *p* < 0.05), and 30 m (S1, RQ = 0.11 ± 0.004, *p* < 0.05; V1, RQ = 0.10 ± 0.01, *p* < 0.05) compared to P0 in both the S1 and V1 (Figure 4C). The data show a comparable age-related alterations in both the protein expression and mRNA levels of STMN in the S1 and V1.

#### Immunohistochemistry

The focus of STMN expression was in neuronal cells at the neonatal stage of age and in mircoglial cells at elderly stage of age. STMN was detected in close association with the nucleus in NF-200-positive neuronal cells in both cortical regions (Figures 4D–K). In contrast, colabeling of STMN was found in the cytoplasm in both GFAP-positive cortical glial cells and OX-42-positive microglia.

#### Neuronal staining

STMN-positive neuronal cells were in found in neonatal rat cortices (S1, 89.1 ± 7%; V1, 88 ± 6.6%; Figures 4L–P). Its colabeling rate decreased massively over life-time until late adulthood (e.g., 18 m; S1, 13.7 ± 3%, *p* < 0.05; V1, 13.7 ± 4.5%, *p* < 0.05) compared to P0. A decrease of these STMN-positive cells compared to P0 and adult stages (12 m, 18 m) was found in the S1 also in elderly stages (30 m: 4.9 ± 2%, *p* < 0.05). An increase in STMN staining in neuronal cells was detected in the V1 at P30m (35.8 ± 7.8%) compared to 18 m (40.2 ± 6.2%, *p* < 0.05) (Figure 4L).

#### Glial staining

A significant difference in colabeling of STMN and GFAP-positive glial cells was found in S1 (30.1 ± 8%) compared to V1 (88 ± 6.6%, *p* < 0.05) at neonatal stages (Figures 4Q–U). A decrease in the proportion of the cells in which STMN and GFAP were costained in both the S1 (9.5 ± 4.5%, *p* < 0.05) and V1 (6.6 ± 1.8%, *p* < 0.0.05) in the adult stages (18 m) compared to P0. At P30m, the proportion of STMN-positive glia cells show a tendency to increase in the S1 (24.7 ± 10%, *p* = 0.5) compared to P0 (Figures 4Q,V–Y). A further decrease in the colabeling rate of STMN with GFAP-positive glial cells was found in the V1 (6 ± 1.9%, *p* < 0.05) compared to P0.

#### Microglial staining

Noticeable colabeling of STMN and OX-42-positive microcglial cells was detected in both S1 (46.6 ± 10.9%) and V1 (23.1 ± 5.2%, *p* < 0.05) at P0 (Figure 4Z). The proportion of STMN-positive microglial cells remained unchanged in the S1 until the elderly stage of age (e.g., 30 m: 54.5 ± 9.5%, *p* = 0.4), with exception of early adulthood (12 m: 62.6 ± 11.6%) compared to advanced adult stages of age (18 m: 37.6 ± 13.7%, *p* < 0.05) (Figures 4AA–AD). In contrast, an increase of proportion of costaining was observed in the V1 (6 m: 43.4 ± 6%, *p* < 0.05; 18 m: 40.2 ± 6.1%, *p* < 0.05; 30 m: 62.1 ± 11.7%, *p* < 0.05) compared to P0 (Figure 4Z).

## Discussion

We examined the expression of Prx, SNCB, DJ-1 and STMN during the postnatal maturation and aging in two areas (S1 and V1) of the cerebral cortex in rats (Böhm et al., 2013). Although the work presented here, including quantitative approaches toward the protein expression has limitations, to the best of our knowledge this has not been done previously and this is the first study to describe the expression of these age-related proteins in morphologically and functionally different cortical regions. The principal findings of the study are as follows:

1. The S1 and V1 share in common the postnatal maturation and age-related proteins Prx, SNCB, DJ-1, and STMN, which have previously been described in the retina.
2. Pyramidal neurons and glial cells exhibit a decrease in Prx expression in both the S1 and V1 in an age-related context.
3. SNCB expression increased in V1 pyramidal neurons during adulthood, whereas it remained unchanged over life-time in the S1.
4. DJ-1 expression decreased continuously in the S1 pyramidal neurons during the measured life-time, whereas its expression increased to neonatal levels in the elderly V1.
5. A massive reduction in STMN expression has been found in neuronal cells and glial cells in both the S1 and V1.

Studies in the last decades regarding cortical development and aging have been performed mainly with tissue obtained from human and nonhuman primates (Huttenlocher, 1990; Rakic et al., 1994; Elston and Rosa, 2006; Bianchi et al., 2013; Oga et al., 2013). Few of these studies have revealed differences in the cortical development of rodents compared to primates, although marked differences have been demonstrated in pyramidal cell structure in homologous cortical areas between primates and rodents (Elston and Manger, 2014). Studies comparing mouse, rhesus macaque monkey, and human brains have revealed divergence in the expression patterns of major genes (Loerch et al., 2008). For example, up-regulation of genes participating in neuronal functions was found during aging in mice, but these genes were down-regulated during aging in humans. Given the assumption of an evolutionary shift between the lineage of rodents and humans, this finding may be attributed to specific human neurodegenerative disorders such as AD. The data presented in this study were assessed using the brains of Sprague-Dawley rats. These laboratory animals are easy to access and provide “comparable” life events due to their short lifespan relative to humans. Moreover, the quantification of the subset of cortical cells was performed with semi quantitative methods and not with high-performance stereological methods. Nevertheless, interesting alterations in the protein expression in rats due to development and aging were detected in the selected cortical regions. We will briefly review what is already known in rodents, humans and nonhumans. Then, we will discuss in detail the selected proteins and regional differences in their patterns of expression in the two cortical areas of interest.

Genetic and epigenetic assumptions, area-specific factors, and the function of its projections influence pyramidal cell structure and create regional variations in pyramidal cell phenotypes in the primate cerebral cortex (Vercelli and Innocenti, 1993; Elston et al., 1996, 2011a; Matsubara et al., 1996; Elston and Rosa, 1997, 1998, 2006; Elston, 2000; Jacobs et al., 2001; Elston and Rockland, 2002; Benavides-Piccione et al., 2006; Bianchi et al., 2013; Oga et al., 2013; Sasaki et al., 2014). A physiological loss of neuronal cells and synapses is likely caused by a failure of developing neurons to find targets for innervation. This observation is missing in complex neuronal systems such as the human neocortex, whereas a 30% loss of cortical neurons has been observed in rodents (e.g., in the mouse) (Heumann et al., 1978; Heumann and Leuba, 1983). There is also little evidence for overproduction of synapses in rodents, and the synaptic density in the rat brain reaches a maximum at about postnatal day 35, which is less than 10% greater than in the adult (Aghajanian and Bloom, 1967). During the first postnatal month in cats, glial cells may influence the development of axons, and both microglial and astrocytes participate in the shaping of the callosal cortical maps to the level of the V1 in mammals (Rochefort et al., 2002).

Physiological aging in the mammalian brain is characterized by several interrelated morphological and metabolic changes. Recent morphological studies have revealed considerable variations in different parts of the brain, including the human cerebellum and the cerebral cortex of rhesus monkeys (Nandy, 1981). Physiological aging is not associated with significant neuronal loss in the human or macaque neocortex (Peters et al., 1994; Pakkenberg and Gundersen, 1997). Instead, age-related cognitive decline is thought to result from more subtle synaptic alterations (Morrison and Hof, 1997). Qualitative observations of dendritic spine reduction have been made in the aging cortex of humans, nonhuman primates, mice and rats, which are consistent with reports of age-related decreases in synapses in different cortical regions (Lee et al., 2000; Fraser et al., 2005; Yankner et al., 2008; Stranahan et al., 2012).

Age-related metabolic changes include a global reduction in the brain’s energy requirements and decreases in cerebral blood flow and glucose utilization (Chugani et al., 1991). Recent studies exploring age-related changes in dendrites and dendritic spines in the frontopolar and occipital regions of the human neocortex indicate that different cortical areas in primates do not age in a uniform manner. For example, the prefrontal cortex appears to be more susceptible to aging than sensory regions such as the occipital cortex (Azari et al., 1992; Eberling et al., 1995; Jacobs et al., 1997). The prefrontal cortex exhibits a higher metabolism and regional cerebral blood flow in the normal (resting) state than other cortical areas (Roland, 1984). Life-time changes in cortical metabolism parallel the age-related variations in synaptic densities observed in the frontal cortex (e.g., S1), but they correlate less with changes in synaptic density in the V1 (Jacobs et al., 1997). Aging is also a risk factor for progressive brain disorders in which neuroinflammation plays a prominent role. Coordinated changes in gene transcription cascades underlie changes in synaptic, neurotrophic, and inflammatory phenotypic networks during brain development, maturation and aging. Early postnatal changes in gene expression are related to neuronal, glial, and myelin growth, and synaptic pruning events, while late aging is associated with proinflammatory and synaptic loss (Primiani et al., 2014). Thus, the distribution and morphology of astrocytes and microglial cells changes with age (Rochefort et al., 2002).

### Peroxiredoxin

Prx was expressed in both the rat S1 and V1 throughout the ages examined. In the present study, the protein levels of Prx did not change significantly in the rat cortex during aging, although a slight decrease in its mRNA was observed in both cortical regions. The expression of Prx have been recently studied in the human, murine, and (in the case of Prx-1) rat brain (Sarafian et al., 1999; Mizusawa et al., 2000; Wang et al., 2003). Prx are presumably involved in various cellular reactions, such as cellular defense against reactive oxygen species (ROS), receptor signaling, gene regulation, and apoptosis (Jim and Jeang, 1999; Singh and Shichi, 2001). They are functioning mainly to support cells, including oligodendrocytes and Schwann cells, probably protecting them against oxidative stress (Mizusawa et al., 2000). Prx-1 is associated with glial cells, and particularly with oligodendrocytes and neurons (Mizusawa et al., 2000; Aon-Bertolino et al., 2011). Prx-2, -3, -4, and -5 have been detected in projection neurons in the human brain (Sarafian et al., 1999; Aon-Bertolino et al., 2011). Loss of mitochondria in CA1 pyramidal neurons has been detected in Prx-2-defcient mice due to the functioning of the extracellular signaling kinases pathway (Kim et al., 2011). Neuroglial localization of Prx-6 has been detected in mouse and human brains (Wang et al., 2003; Aon-Bertolino et al., 2011), with a high degree of specificity for this cell type.

An age-dependent diversification of the cell types expressing Prx was detected. An association between Prx and neuronal, glial, and microglial cells was found in both the S1 and V1 of the neonatal rat brain. A reduction in Prx labeling of both neuronal and glial cells during aging suggests a loss of mechanisms for responding to oxidative stress in major cell types in the cortex. The age-related changes in the expression of Prx and the associated cell types detected in the present study are probably associated with changes in the vulnerability of cortical neurons to oxidative stress. Brain samples from elderly individuals have revealed decreased Prx-2 protein levels, which may induce increased oxidative stress in the aging brain (Chen et al., 2003; Aon-Bertolino et al., 2011). Decreased Prx-3 expression was detected in regions known to be specially affected in AD, Down’s syndrome, and PD (Kim et al., 2001; Krapfenbauer et al., 2003). We observed a decrease in Prx expression in pyramidal cortical cells in the V1 after 6 months of life, compared to a decrease after 12 months in the S1. This finding could be associated with the reported slower progress of dendritic development and increase in synaptic density in the S1 compared to the V1 (Huttenlocher and Dabholkar, 1997; Jacobs et al., 1997).

The increase of microglial cells positive stained for Prx during aging suggests an increased activation of immunoassociated cell types in the cortical regions. This observation concurs with the reported neuronal loss of up to 30% and overproduction of synapses during rodent brain maturation between birth and adulthood. These changes roughly parallel the age-related variations in synaptic densities observed in the S1, but are less well correlated with changes in synaptic density in the primary V1 (Heumann et al., 1978; Heumann and Leuba, 1983; Huttenlocher and Dabholkar, 1997). Moreover, an earlier increase in the proportion of Prx-positive microglial cells was found in the V1 compared to the S1, where this increase in microglial Prx staining began in the adult ages. The diversity of Prx-positive pyramidal cells and microglia in different cortical areas may reflect this difference in metabolic demand, including metabolic and oxygen stress in the S1 and V1.

Comparison of Prx expression relative to subsets of brain cells revealed a shift from neurons and glia toward microglia. However, comparison between the areas examined revealed that the expression and colabeling profiles were not comparable between the two, suggesting that the proteins show aspects of known cortical development steps reflecting regional features. A sole association with visual processing cannot be concluded. The uniform expression of Prx over the frontal and occipital lobes may be associated with the vulnerability of the brain to oxidative stress in comparison to other organs due to its high oxygen utilization, high iron content, presence of unsaturated fatty acids, and reduced activities of detoxifying enzymes such as superoxide dismutase, catalase, and glutaredoxins (Dringen, 2000; Capani et al., 2001; Rodríguez et al., 2005). The disturbance of redox homeostasis, low levels of glutathione, and increased production of ROS and peroxynitrite have been described for several CNS disorders, such as perinatal asphyxia (Capani et al., 2001), stroke (Eliasson et al., 1999), focal traumatic brain injury (Singh et al., 2006), and numerous neurodegenerative disorders including AD, PD, multiple sclerosis, and amyotrophic lateral sclerosis (Bains and Shaw, 1997; Torreilles et al., 1999). Lastly, the localization of Prx in neuronal cells in neonatal, and microglial cells in aged cortical regions concurs with the localization reported in the aging retina (Böhm et al., 2013).

### Beta-synuclein

We detected an increase expression of SNCB within both the S1 and V1 over the life-time of rats. The family of cytoplasmic synuclein proteins that comprises SNCA, SNCB, and gamma-synuclein (SNCG) is thought to function in synaptic vesicles and neurotransmission, and neuronal plasticity. SNCA and SNCB are highly homologous proteins, and are colocalized in presynaptic nerve terminals in the CNS. In contrast, SNCG is expressed primarily in the PNS (Hashimoto et al., 2001; Sung and Eliezer, 2007). SNCB may elicit neuroprotective functions, e.g., the neurotoxic response in 6-hydroxydopmaine-affected TSM-1 (twin sensillum of margin 1) neurons (Hashimoto et al., 2001, 2004; Park and Lansbury, 2003; Tsigelny et al., 2007). It has been shown that SNCB decreases the proapoptotic gene *p53* (da Costa et al., 2003). Furthermore, SNCB activates the Akt signaling pathway in rotenone-affected tissue culture B103 cells (Hashimoto et al., 2004). Hashimoto suggested that the SNCB activation of Akt resulted in Mdm2 (mouse double minute 2 homolog) phosphorylation, which in turn inactivates *p53*. This mechanism may promote neuroprotection against toxins (Hashimoto et al., 2004). Finally, SNCB protects the CNS against the toxic effects of SNCA overexpression in *tg* mice (Spillantini et al., 1997), in which SNCB overexpression results in increased Akt pathway activity, suggesting that the phosphatidylinositide-3-kinase signaling pathway is a potential therapeutic target for PD via SNCA aggregation.

The expression pattern of SNCB varied considerably in the rat cortices throughout the life-time. It was expressed in both the V1 and S1, beginning at 6 m. In contrast to S1, early expression of SNCB was detected in the neonatal V1. In neonatal cortices, SNCB was detected in close association with neuronal and glial cells; it was less clearly correlated with microglial cells. The increased SNCB expression after birth may reflect the fact that the synaptic density in the rat brain reaches a maximum at about postnatal day 35, which is only less than 10% greater than the adult value (Aghajanian and Bloom, 1967). As yet we have no direct explanation for the early expression of SNCB in the V1. It may be associated with the increased requirements for early postnatal maturation in the S1 in contrast to the sparsely developed neonatal visual system of rats.

An age-related divergence in the increase in SNCB-positive pyramidal cells was found in both adult rat cortices in this study (i.e., S1 and V1). An increased association between SNCB and pyramidal neurons was found in V1 beginning at the adult stage of life. In contrast, the S1 exhibited mainly SNCB colocalized with NF-200-positive neuronal cells in the elderly rats. We presume that the earlier increase in SNCB expression by pyramidal neurons could reflect the lower vulnerability of the V1 to aging in contrast to the sensory cortex, possibly due to the reported association with a higher metabolic rate and regional blood flow in the S1.

We found comparable levels of SNCB expression in the S1 and V1 and in the aging retina (Böhm et al., 2013). In contrast to the strong association with the synapse-rich retinal layers, SNCB in the cortex appeared to be associated with neonatal neuronal cells and aged glial cells.

### DJ-1

DJ-1 is expressed in many tissues, including the brain without any preference to a single functional system or anatomical area (Nagakubo et al., 1997; Bader et al., 2005). In this study, DJ-1 expression was found in both the V1 and S1 throughout the life-time of rats. In the V1 DJ-1 expression increased up to the late adult age and then regressed to neonatal levels, that in the S1 increased continuously from P0 to the senile age stages. DJ-1 plays a cardinal role in maintaining mitochondrial function and is reported to possess neuroprotective properties by limiting oxidative damage (Moore et al., 2005). DJ-1 can enhance antioxidant systems and promote antioxidant mechanisms (Liu et al., 2008; Blackinton et al., 2009). Moreover, it is involved in the functioning of the ubiquitin-proteasome system, which reduces the accumulation of toxic protein substrates. Mitochondrial dysfunction (and the associated oxidative stress) and altered functioning of the ubiquitin-proteasome system are considered important factors in familial and sporadic forms of PD. Importantly, it is widely accepted that impairments of these mechanisms are common denominators of neurological disorders in general, and in particular AD and multiple sclerosis, and may already have occurred in the early stages of these diseases (Moreira et al., 2005; Lassmann and van Horssen, 2011; van Horssen et al., 2011; Wilhelmus et al., 2012). Neverless, DJ-1 may operate at the intersection of environmental stressors and aging (Chen et al., 2014).

DJ-1 is expressed in neurons with different neurotransmitters and in all glial cell types, such as astrocytes, microglia and oligodendrocytes. In a number of cell lines, DJ-1 is associated with microtubules and localizes to both the nucleus and the cytoplasm (Hod et al., 1999; Bader et al., 2005). The neuronal and synaptic expressions of DJ-1 in primate subcortical brain regions suggest a physiological role for DJ-1 in the survival and/or function of nigrostriatal neurons (Olzmann et al., 2007). The expression characteristics of DJ-1 in pyramidal neurons reported herein for the S1 and V1 are comparable to other examined stress-related proteins, like Prx. The proportion of DJ-1 in NF-200 positive neurons decreased gradually from the day of birth with aging in the S1, whereas it increased to adulthood in the V1. The loss of associated DJ-1 in neuronal cells may indicate a loss of protective factors in the aging brain. A reduced expression of DJ-1 in glial cells was found in both of the cortical areas examined in young adulthood compared to older brains. Recent studies described the abundantly-expression of DJ-1 in PD astrocytes (Bandopadhyay et al., 2004; Neumann et al., 2004; Rizzu et al., 2004; Mullett et al., 2009). This may represent an attempt by astrocytes to protect themselves, and surrounding neurons, against disease progression. DJ-1 is over-expressed in astrocytes enhancing their neuroprotective capacity against rotenone and other pesticides *in vitro*. DJ-1 knock-down astrocytes were impaired in this capacity. DJ-1 modulate the release of soluble factors by astrocytes (Mullett and Hinkle, 2009, 2011). The prevalence of DJ-1 expression in microglia increased with aging in both the S1 and V1. DJ-1-deficient microglia had increased monoamine oxidase (MAO) activity that resulted in elevation levels of neurotoxic secreted factors, including intracellular ROS, nitric oxide, and pro-inflammatory cytokines (Trudler et al., 2014). These findings suggest an increased association between DJ-1 expression and cells associated with the innate immune system in the cortex.

The localization of DJ-1 in the retina also varies over life-time. In newborn rats, DJ-1 has been found in the germinative ganglion cell layer, and after P16 and marginally at P23 and P60 in the inner plexiform layer (Böhm et al., 2013). Although the exact role of DJ-1 in the retina is not clear, its main role may be in the early retinal maturation (Haniu et al., 2006; Finnegan et al., 2008). However, these data indicate comparable expressions of DJ-1 in visual-specific neuronal regions during aging.

Given the role of DJ-1 in maintaining mitochondrial function and reducing oxidative stress, this pattern of expression may reflect the lower vulnerability of the V1 to aging and oxidative stress compared to the S1. The presented findings reveal a difference in the expression of DJ-1 in various cortical regions and cortical cell types show over the life-time in rats.

### Stathmin

Our data show a dramatic decrease in STMN in both the S1 and V1 beginning at 6 m; thereafter, the expression of STMN remained stably low until 30 m. These observations are in general agreement with previous studies, which have found STMN in the late embryonic and early postnatal phase of cerebral development. During rat development, the highest expression of stathmin family proteins is from late embryogenesis until a week after birth, when dendrite formation, axon guidance and synaptic in the developing CNS are most dynamic and has mainly found in the cortex and nucleus accumbens (Ozon et al., 1999; Hayashi et al., 2006). Proteins of the stathmin family proteins are expressed in different cell populations, including neurons and glial cells (Ozon et al., 1999; Charbaut et al., 2001). Stathmin has been implicated in growth, differentiation and cell cycle control (Sobel, 1991; Schubart et al., 1996). Stathmin is involved in neural development, differentiation, plasticity, learning, degeneration and aging (Mori and Morii, 2002; Nelson et al., 2004). These studies are in agreement with the neonatal findings of this study, that STMN was mainly found in NF-200-positive neuronal cells. Protein and mRNA levels of STMN have also been found in the developing, maturating, and adult CNS in several species, including chicken, mouse, rat and in post-mortem human brains from schizophrenia and AD patients (Ozon et al., 1999; Hayashi et al., 2006; Finnegan et al., 2008). A decrease of forced expression of stathmin in response to lesioning of the adult rat cortex has been found with age (Hayashi et al., 2006). This may be in accordance to the findings of a massive decreased association between STMN and pyramidal neurons in both the S1 and V1 together with a decrease of neuronal plasticity during life-time until elderly stages of age (Hayashi et al., 2006).

Recent studies indicate a regulatory role of STMN during inflammtation and repair in the adult CNS (Bsibsi et al., 2010). Shen et al. reported about a activated serine–threonine kinase interacting stathmin (KIS) due to spinal cord injury, which interact and posporylate stathmin inducing cell cycle progression of glial cells, especially microglia and astrocytes (Petrovic et al., 2008; Shen et al., 2008). Further studies found a colocalization of stathmin with TLR3 on astrocytes, microglia, and neurons in multiple sclerosis-affected human brains (Bsibsi et al., 2010). Nigrostrial dopimergic neurodegeneration and the expression levels of STMN were significantly depentend on microglial activation (Singh et al., 2011). In the present study, an increase in STMN-positive microglial cells in V1 was found in contrast to unchanged costainings in the S1 over life-time. Taken together, the observations in the present study are probably in accordance with the less vulnerability of the V1 compared to S1 regarding to oxidative stress and aging. We recently described a strong expression of STMN in the neonatal rat retina, with a subsequent decrease during retinal maturation, and no expression after 2 months of life. During embryonic development and early maturation of the retina, STMN was found in both the IPL and OPL (Böhm et al., 2013). The expression profile of STMN appears to be correlated with neuronal differentiation and plasticity in the younger retina; its detection in the retinal plexiform layers in the mature retina might be correlated with ongoing synaptic plasticity in the adult tissue (Nakazawa et al., 2000). STMN expression in the aging S1 and V1 in the present study exhibited patterns that were comparable with that of the aging neuroretina. It appears that there is an almost identical age-related regulation of STMN in the cortex and retina. To the best of our knowledge, the present study is the first to reveal an association between STMN and visual associated areas in the CNS.

## Conclusions

The alterations of the detected proteins in both the retina and cortex may be associated with generalized mechanisms in postnatal development, maturation and aging. Distinct cortical areas were found in this study to exhibit differential patterns of expression of Prx, SNCB, DJ-1, and STMN. The alterations in Prx and DJ-1 with aging are likely associated with impairment of the oxidative-stress-regulating process. If a similar increase in SNCB expression also occurs over the life-time in the human brain, it may be related to neurodegenerative diseases such as AD. The massive reduction in STMN may be associated with loss of neuronal plasticity during CNS aging. Summarized in a comparative context, the age-related expression profile of proteins within the V1 appears to be similar to that in the retina. The results support the hypothesis of Elston, that developmental and maturation mechanisms and area-specific factors have a significant effect on the cell morphology of pyramidal neurons, the process of forming a neuronal network, and synaptic densities in different cortical areas (Elston, 2002, 2007). More over, the observed alterations in protein expression over life-time concurs with developmental, maturational and age-related changes in oxidative stress response, metaboblic impairments and loss of the neuronal network. Further studies are needed to confirm the protein changes reported herein in other species, and would contribute to a better understanding of the mechanisms underlying the senescence that may predict potential neurodegeneration.

## Conflict of interest statement

The authors declare that the research was conducted in the absence of any commercial or financial relationships that could be construed as a potential conflict of interest.

## Footnotes

^1^http://imagej.nih.gov/ij/
